# Actigraphy assessments of circadian sleep-wake cycles in the Vegetative and Minimally Conscious States

**DOI:** 10.1186/1741-7015-11-18

**Published:** 2013-01-24

**Authors:** Damian Cruse, Aurore Thibaut, Athena Demertzi, Julia C Nantes, Marie-Aurélie Bruno, Olivia Gosseries, Audrey Vanhaudenhuyse, Tristan A Bekinschtein, Adrian M Owen, Steven Laureys

**Affiliations:** 1 Brain and Mind Institute, University of Western Ontario, 1151 Richmond Street, London, ON N6A 3K7, Canada; 2 Coma Science Group: Department of Neurology and Cyclotron Research Centre, University Hospital and University of Liège, Allée du 6 août n° 8, Sart Tilman B30 - 4000, Belgium; 3 Cognition and Brain Sciences Unit, Medical Research Council, 15 Chaucer Road, Cambridge, CB2 7EF, UK

**Keywords:** Vegetative State, Minimally Conscious State, circadian rhythms, sleep, actigraphy

## Abstract

**Background:**

The Vegetative and Minimally Conscious States (VS; MCS) are characterized by absent or highly disordered signs of awareness alongside preserved sleep-wake cycles. According to international diagnostic guidelines, sleep-wake cycles are assessed by means of observations of variable periods of eye-opening and eye-closure. However, there is little empirical evidence for true circadian sleep-wake cycling in these patients, and there have been no large-scale investigations of the validity of this diagnostic criterion.

**Methods:**

We measured the circadian sleep-wake rhythms of 55 VS and MCS patients by means of wrist actigraphy, an indirect method that is highly correlated with polysomnographic estimates of sleeping/waking.

**Results:**

Contrary to the diagnostic guidelines, a significant proportion of patients did not exhibit statistically reliable sleep-wake cycles. The circadian rhythms of VS patients were significantly more impaired than those of MCS patients, as were the circadian rhythms of patients with non-traumatic injuries relative to those with traumatic injuries. The reliability of the circadian rhythms were significantly predicted by the patients' levels of visual and motor functioning, consistent with the putative biological generators of these rhythms.

**Conclusions:**

The high variability across diagnoses and etiologies highlights the need for improved guidelines for the assessment of sleep-wake cycles in VS and MCS, and advocates the use of actigraphy as an inexpensive and non-invasive alternative.

## Background

The Vegetative State (VS) or Unresponsive Wakefulness Syndrome (UWS [1]) is thought to reflect the dissociation of the two primary components of consciousness - awareness and wakefulness [2,3]. A common tool for the assessment of awareness is the Coma Recovery Scale Revised [4] (CRS-R) which includes subscales designed to assess a range of functions, including auditory, visual, motor, verbal, communication and arousal. A brain-injured patient is considered to possess awareness if they produce non-reflexive responses to stimulation, such as tracking an object that is moving in front of the eyes, or following a verbal command. Patients in the VS do not produce non-reflexive behavior and are, therefore, considered to lack awareness [5,6]. Patients in the Minimally Conscious State (MCS) exhibit some reproducible but inconsistent signs of awareness, although communication remains absent [6,7].

Wakefulness, on the other hand, is thought to be preserved in both VS and MCS patients. According to the standards for VS and MCS outlined by the Multi-Society Task Force for Permanent Vegetative State [5] and the Royal College of Physicians [6], 'wakefulness' refers to the presence of typically cycling periods of eye-closure and eye-opening that give the appearance of sleep-wake cycles. While a great deal of behavioral and neuroimaging research has focused on the assumption of unawareness in these patients [4,8-10], very little is known regarding the assumption of preserved sleep-wake rhythms.

A typical sleep-wake cycle follows a circadian rhythm, with a period of between 19- and 28-hours [11]. Electroencephalography (EEG), in combination with other physiological measures as part of polysomnography, is the gold-standard approach for the assessment of sleep-wake cycles [12]. However, the results of the limited EEG investigations of circadian sleep-wake cycling in VS and MCS patients are inconsistent with the assumption of preserved wakefulness. Landsness *et al. *[13] observed sleep-wake-like changes in the EEG of six MCS patients across one day, while the EEG of five VS patients remained unchanged between periods of eye-opening and eye-closure. Isono *et al. *[14] also reported an absence of EEG sleep-wake changes in 4 out of 12 VS patients. High variability has also been observed in other physiological circadian rhythms in VS and MCS, including body temperature and hormone levels [15,16], blood pressure and heart rate [16,17], and sleep-related erections [18]. Circadian-like variations in arousal have also been reported in both VS and MCS patients, as indexed by fluctuating behavioral abilities across the day [19]. Bekinschtein *et al. *[20] observed well-formed circadian rhythms in the body temperatures of two VS patients with traumatic brain injuries (TBI), but absent rhythms in three VS patients who had sustained non-traumatic brain injuries (non-TBI), indicating the potential relationship between etiology and circadian rhythms. It appears, therefore, that, contrary to the diagnostic guidelines describing these conditions, a great deal of variability exists both within and across VS and MCS patient groups with regard to the relative preservation of circadian rhythms.

An indirect and inexpensive approach to detecting circadian sleep-wake cycles from large numbers of patients is wrist actigraphy, in which a wrist-mounted device is used to record the frequency and amplitude of motor activity [12]. This method is known to correlate well with polysomnographic measurements of sleep and wakefulness in healthy individuals, as well as non-ambulatory patients, such as those with C5 to C7 tetraplegia [21-23]. A number of algorithms have been developed in order to produce minute-to-minute estimations of sleeping/waking from short-term variations in actigraphy data in healthy individuals. Broadly, these algorithms judge an individual to be awake or asleep at a given sample point by weighting the amount of movement in a number of preceding sample points by a set of predefined constants. Such approaches have reported between 88 and 97% concordance with polysomnography in healthy individuals (see [21] for a full review). However, none of these approaches have been validated with VS or MCS patients by means of concurrent polysomnography and actigraphy recordings. Nevertheless, a circadian sleep-wake rhythm - that is, more activity during waking hours and less activity during sleeping hours - can be readily identified from raw actigraphy recordings, and makes fewer assumptions than these un-validated algorithms (for example, [24,25]). In the only article to report actigraphy-based assessments of sleep-wake rhythms in VS, Bekinschtein *et al. *[26] described a greater deterioration in the circadian rhythmicity evident in the actigraphy of one VS patient relative to an MCS patient. De Weer *et al. *[27] also reported day-night variation in the amount of movement (as measured by actigraphy) in two TBI MCS patients, but not in a non-TBI MCS patient. However, in neither of these studies was circadian rhythmicity examined statistically.

In order to investigate the relative preservation of circadian sleep-wake rhythmicity in patients in the VS and MCS, we recorded wrist actigraphy from 55 patients (18 VS, 37 MCS) across four days, and subjected the data to cosinor rhythmometry analyses (see Methods), a standard statistical approach for circadian rhythm identification. By definition, all of these patients are considered to possess circadian sleep-wake cycles [5-7]. In keeping with the studies described above, however, we expected to see variability in the extent to which circadian sleep-wake rhythms were preserved across patients as a function of etiology (TBI vs. non-TBI) and diagnosis (VS vs. MCS). We also predicted significant relationships between the behavioral profiles of these patients - as indexed by their CRS-R subscales - and the relative preservation of their circadian sleep-wake rhythms.

## Methods

### Patients

Fifty-five patients were recruited from the University Hospital of Liège, Belgium. Actigraphy recordings were made for at least four days. All patients were VS or MCS. During their admission, all patients were manually turned in their beds four times per day. No patient had skin pressure sores that required more frequent manual turning. No patient required mechanical ventilation. All patients were admitted as part of the same research protocol, and completed the same tasks across each day, for example, behavioral tests, positron emission tomography (PET), and magnetic resonance imaging (MRI). Across their admission, all patients were assessed multiple times with the CRS-R [4]. The highest CRS-R score and diagnosis across this period are shown in Table 1, along with other demographic information. In total, 18 VS patients (mean age 38.0, SD 14.8; 7 TBI) and 37 MCS patients (mean age 35.7, SD 15.2; 24 TBI) contributed data to the study. There was no significant difference in the proportions of each etiology contributing to the VS and MCS groups. Two two-way ANOVAs with factors of diagnosis (VS, MCS) and etiology (TBI, non-TBI) conducted on age (in years) and months *post-ictus *revealed only a reliable main effect of etiology on age (F(1, 51) = 10.363, *P *<.01) reflecting the older average age of non-TBI patients. Informed consent was obtained from the patients' surrogate decision makers. The Ethics Committee of the University and University Hospital of Liège provided ethical approval for the study.

**Table 1:** Demographics and circadian rhythm fits for all patients.

Patient ID	Gender	Age (Years)	Post-Ictus (Months)	Diagnosis	Etiology	CRS-R	Mesor	Acrophase	Amplitude	Sig. Fit?
1	M	53	40	MCS	Non-TBI	11	9.56	16:57	6.70	Yes
2	M	31	22	MCS	Non-TBI	13	8.67	21:44	10.46	Yes
3	W	30	78	MCS	Non-TBI	9	15.84	15:45	13.58	Yes
4	M	31	16	MCS	Non-TBI	7	5.51	19:27	4.63	Yes
5	M	27	50	MCS	Non-TBI	9	31.01	15:43	9.94	No
6	W	36	17	MCS	Non-TBI	13	21.25	19:10	15.87	Yes
7	M	34	35	MCS	Non-TBI	12	26.40	17:35	28.34	Yes
8	W	63	3	MCS	Non-TBI	13	6.32	16:36	6.77	Yes
9	M	57	12	MCS	Non-TBI	7	12.89	17:26	9.40	Yes
10	M	66	2	MCS	Non-TBI	10	34.18	22:25	26.00	Yes
11	M	11	48	MCS	Non-TBI	13	32.40	19:05	35.79	Yes
12	W	43	3	MCS	Non-TBI	6	9.12	17:49	8.76	Yes
13	W	34	256	MCS	Non-TBI	12	20.63	18:40	15.36	Yes
14	M	30	106	MCS	TBI	14	49.20	20:18	41.91	Yes
15	W	21	1	MCS	TBI	10	17.23	17:57	12.52	Yes
16	M	46	17	MCS	TBI	11	42.80	22:46	48.47	Yes
17	M	30	27	MCS	TBI	10	20.71	20:12	17.98	Yes
18	M	30	13	MCS	TBI	9	54.24	17:16	22.60	Yes
19	M	24	10	MCS	TBI	10	114.11	18:50	65.35	Yes
20	W	75	9	MCS	TBI	9	8.57	17:06	9.55	Yes
21	W	34	99	MCS	TBI	12	18.22	15:46	6.64	No
22	W	27	41	MCS	TBI	11	15.02	23:05	16.01	Yes
23	M	24	88	MCS	TBI	11	18.54	19:49	14.62	No
24	M	44	287	MCS	TBI	9	5.25	19:37	4.83	Yes
25	W	30	4	MCS	TBI	9	10.41	17:29	13.41	Yes
26	M	34	33	MCS	TBI	8	27.22	18:30	27.89	No
27	M	23	10	MCS	TBI	10	8.47	16:38	7.74	Yes
28	M	27	37	MCS	TBI	13	40.74	20:53	34.18	Yes
29	M	61	4	MCS	TBI	10	21.80	21:41	19.72	Yes
30	M	24	24	MCS	TBI	11	88.98	22:18	56.45	No
31	M	23	66	MCS	TBI	16	14.09	17:24	18.96	No
32	M	21	38	MCS	TBI	8	10.09	15:55	7.06	Yes
33	M	30	109	MCS	TBI	10	35.15	20:26	21.87	Yes
34	W	24	21	MCS	TBI	10	15.45	18:58	18.08	Yes
35	M	36	4	MCS	TBI	11	6.49	16:10	5.53	Yes
36	M	65	22	MCS	TBI	7	11.30	16:13	15.14	Yes
37	M	21	5	MCS	TBI	7	9.69	13:41	7.90	Yes
38	W	66	0	VS	Non-TBI	3	4.56	15:43	5.24	Yes
39	M	35	220	VS	Non-TBI	7	11.01	19:20	10.24	Yes
40	M	30	24	VS	Non-TBI	6	25.15	15:28	14.67	Yes
41	W	48	15	VS	Non-TBI	5	8.50	17:14	10.53	Yes
42	W	67	45	VS	Non-TBI	5	15.46	18:29	12.23	Yes
43	M	53	1	VS	Non-TBI	5	9.53	16:53	7.92	Yes
44	M	34	17	VS	Non-TBI	7	5.68	17:43	2.79	Yes
45	W	41	56	VS	Non-TBI	5	14.39	17:35	14.49	Yes
46	W	48	4	VS	Non-TBI	4	4.78	20:02	3.44	No
47	M	48	30	VS	Non-TBI	6	2.31	17:19	2.03	Yes
48	M	36	66	VS	Non-TBI	5	7.34	17:40	6.88	Yes
49	M	34	43	VS	TBI	6	10.84	13:47	7.87	Yes
50	W	30	18	VS	TBI	4	6.49	16:56	8.07	Yes
51	M	21	7	VS	TBI	7	8.58	18:15	8.29	Yes
52	M	35	290	VS	TBI	8	57.65	23:10	36.56	No
53	M	21	8	VS	TBI	6	9.95	19:22	6.45	Yes
54	M	13	1	VS	TBI	6	5.78	21:24	3.12	No
55	M	25	15	VS	TBI	5	10.02	16:19	9.05	Yes

The final column indicates whether the circadian rhythm fit was significant or not. MCS, Minimally Conscious State; TBI, Traumatic Brain Injury; VS, Vegetative State

### Procedure

Actigraphy recordings were made with a Philips Actiwatch Spectrum (Philips Healthcare, Best, Brabant, The Netherlands) attached to the wrist with the highest range of movement (never the hemiplegic side) for a minimum of four days, sampled in one-minute epochs. In order to normalize across patients, only the first four days of actigraphy data were included in the analyses for those patients who were admitted for longer than four days. The first two hours of data were also excluded to avoid initial artifacts from attachment of the Actiwatch.

### Circadian rhythm analyses

Cosinor rhythmometry analyses [28] were performed on each patient's dataset individually. This approach uses the least squares method to fit a sine wave with a period of 24 hours to the raw actigraphy data [11,12,28]. The rhythmicity of the fit can be described by three parameters: the amplitude, the acrophase, and the mesor. The amplitude of the fit refers to half the distance between the peak and the trough of the fitted wave - in effect describing the amount of movement produced during periods of activity. The acrophase describes the point in the cycle at which activity is maximal. Finally, the mesor (an acronym for *m*idline-*e*stimating *s*tatistic *o*f *r*hythm [28]) describes the rhythm-adjusted mean of the wave, or the value around which the fitted wave oscillates. For equidistant data samples (as employed here), the mesor is equivalent to the arithmetic mean of the fitted wave, or the average amount of activity produced across the recording period. The goodness-of-fit of the wave - that is, the statistical reliability of the circadian rhythm - can also be determined by means of a zero-amplitude F-test [28].

In order to control for over-fitting of noise to the sine wave, this goodness-of-fit *P*-value was subsequently subjected to a permutation test. Specifically, a set of sine waves with periods ranging in 10-minute intervals from 6 hours to 48 hours were fit to the data (excluding rhythms between 19 and 28 hours since these are defined as circadian periods; see Introduction [11]). The *P*-values from these 200 zero-amplitude tests were then used to form a surrogate distribution to test the hypothesis that a 24-hour rhythm does not fit the data better than a non-circadian period. When the goodness-of-fit *P*-value associated with the 24-hour rhythm fell below the smallest 5% of surrogate *P*-values, the circadian rhythm was considered to be significant at *P *<.05.

## Results

A total of 46 out of the whole group of 55 patients (84%) exhibited significant 24-hour rhythms in their actigraphy data after permutation testing. This proportion is significantly lower than the diagnostic expectation that all patients retain significant circadian rhythms (Fisher's Exact Test, *P *<.01). When separated according to diagnosis, 15/18 VS patients (83%) and 31/37 MCS patients (84%) returned circadian rhythms that passed this statistical test. When separated according to etiology, 24/31 TBI patients (77%) and 22/24 non-TBI patients (92%) exhibited circadian rhythms. There was no significant effect of diagnosis or etiology on the proportions of patients exhibiting circadian rhythms (Fisher's Exact Tests, all *P *>.14). While age significantly differed across etiologies, it did not significantly correlate with any of the four rhythmicity variables (mesor, amplitude, acrophase or goodness-of-fit, as indexed by the log-transformed zero-amplitude F-ratio).

### VS versus MCS patients

**Figure 1:**
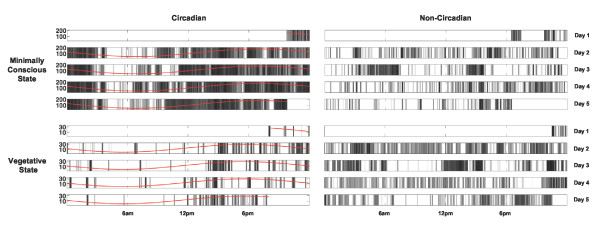
**Actigraphy data from four representative patients**. Each panel shows intensity of activity across each recording day. Red lines indicate the fit of the circadian rhythm. Note the periodic structure of the activity of the two patients with significant rhythms (left), compared with those without (right). Patients 19, 21, 52 and 45 are shown (clockwise from top-left). Log activity data smoothed across five minutes is plotted for clarity of visualization.

Four one-way ANOVAs with diagnosis (VS, MCS) as the factor of interest revealed the main effects of mesor (F(1,54) = 4.441, *P *<.05), amplitude (F(1,54) = 6.819, *P *<.05), and goodness-of-fit (F(1,54) = 16.517, *P *<.001), but not acrophase. Together these reflect the greater average amount of movement across the four days (mesor), the greater amount of movement during periods of activity (amplitude), and greater statistical reliability of the circadian rhythms (goodness-of-fit) of MCS patients relative to VS patients (see Figure 1).

Due to the high inter-correlations between these three significant rhythmicity variables (all absolute r >.33), all three variables were entered into a backward stepwise logistic regression in order to determine their relationships with diagnosis, over and above the effects of the other two variables. This regression retained only goodness-of-fit in the model as a significant predictor (Wald = 10.189, Beta (SE) = -2.043 (.640), *P *<.01), indicating significantly weaker circadian rhythms in VS patients relative to MCS patients, regardless of the amount of movement produced by these patients.

### Traumatic versus non-traumatic brain injury

Four one-way ANOVAs with etiology (TBI, non-TBI) as the factor of interest revealed the main effects of amplitude (F(1,54) = 4.299, *P *<.05) and goodness-of-fit (F(1,54) = 4.226), *P *<.05), but not mesor or acrophase. These effects reflect the greater amount of movement during periods of activity (amplitude) and the greater statistical reliability of the circadian rhythms (goodness-of-fit) of TBI patients relative to non-TBI patients.

As with the analyses across diagnosis, due to the high inter-correlations between the two significant rhythmicity variables, both were entered into a backward stepwise logistic regression in order to determine their relationships with etiology, over and above the effect of the other variable. This regression retained neither variable as a significant predictor, likely due to the weak effects of etiology on these variables (contrast F-values above with those in the analyses across diagnosis).

### Relationship between rhythmicity and behavioral profile

Four backward linear regressions were conducted on the four rhythmicity variables with the six subscales of the CRS-R as predictors. Diagnosis was also included as a predictor since the higher scores on each subscale are also more likely to be associated with MCS and the lower scores with VS. The motor sub-scale was found to significantly predict mesor (F(1,54) = 7.792, *P *<.01, B(SE) = 6.174 (2.212), *P *<.01) and amplitude (F(2,54) = 6.178, *P *<.01, B(SE) = 3.462 (1.453), *P *<.05). The visual sub-scale was found to significantly predict acrophase (F(1,54) = 4.636, *P *<.05, B(SE) = -.108 (.050), *P *<.05), and both the visual and motor subscale together were found to predict goodness-of-fit (F(2,54) = 16.487, *P *<.001, B-visual(SE) = .208 (.055), *P *<.001, B-motor(SE) = .225 (.071), *P *<.005).

## Discussion

On the basis of periodic eye-opening and eye-closure, patients in the VS and MCS are considered to have preserved circadian sleep-wake rhythms [5,6]. However, by means of an indirect measure of sleep-wake rhythmicity - wrist actigraphy - we have shown that a significant proportion of these patients do not exhibit statistically reliable circadian sleep-wake rhythms. The observed variability across patients is consistent with previous smaller studies of circadian rhythmicity in VS and MCS (see Background), and is the first evidence from a large-scale study of sleep-wake cycling using the inexpensive and non-invasive method of wrist actigraphy.

While there was no significant difference in the proportion of patients exhibiting significant sleep-wake rhythms between VS and MCS patients, the goodness-of-fit of the circadian rhythms in the data of MCS patients were significantly higher than those of the VS patients (see Figure 1). This result indicates that the circadian sleep-wake cycles of MCS patients were significantly more statistically reliable than those of VS patients. Importantly, this remained true when taking into account the morphology of the rhythm (that is, its mesor and amplitude), indicating that the effect of diagnosis on the statistical reliability of the circadian rhythms is not driven by simple differences in the amount that a patient moves, but rather reflects differences in the circadian rhythmicity with which this movement occurs.

A master biological clock in the hypothalamic suprachiasmatic nuclei (SCN) is considered to maintain the timing of circadian rhythms. The SCN in turn modulates the activity of the ascending reticular activating system (ARAS) - a circuit of subcortical nuclei responsible for promoting wakefulness (see [29] for a review). One region of the ARAS - the central thalamus - is known to be crucial for the regulation of arousal and has been linked to the disorders of consciousness exhibited by VS and MCS patients [30]. Indeed, the extent of atrophy in this region of the thalamus has been associated with the degree of disability exhibited by these patients [31]. More broadly, greater thalamic atrophy has been observed in VS patients relative to MCS patients using *in vivo *diffusion tensor imaging [32] (DTI). The weaker circadian sleep-wake rhythms observed in the VS patients in the current study are, therefore, entirely consistent with these differential patterns of damage to the thalamus.

Etiology was also shown to have a small effect on the amount that patients moved during periods of activity (amplitude) and the statistical reliability of the circadian rhythm (goodness-of-fit). Similarly, Bekinschtein *et al. *[20] observed reliable circadian temperature rhythms in only TBI VS patients, but not in non-TBI patients, while De Weer *et al. *[27] detected sleep-wake activity changes in only TBI MCS patients. The primary neuropathology associated with TBI is diffuse axonal injury with relative preservation of the cortex, while non-TBI involves more widespread damage to the cortex and basal ganglia [33-39]. The greater impairment of circadian rhythms in non-TBI patients relative to TBI patients reported here is, therefore, consistent with the general patterns of neuropathology associated with the two etiologies. Indeed, mouse models of hypoxic brain injury have been shown to result in impaired sleep-wake cycling [40].

Significant relationships were also observed between the behavioral profiles of the patients - as indexed by their CRS-R sub-scales - and aspects of their circadian rhythmicities. A significant positive relationship was found between the motor subscale and the mesor and amplitude of the rhythm. The motor subscale of the CRS-R is scored from flaccid motor tone at its lowest, to object manipulation and automatic motor responses at its highest (before emergence from MCS). Since wrist movements were used to indirectly measure the circadian rhythms, it is unsurprising that greater amounts of movement exhibited by patients across the recording period (mesor, amplitude) are related to their overall abilities to produce motor output during behavioral assessments. This result suggests the need for caution in the use of actigraphy for assessing circadian sleep-wake rhythms since they rely on motor output for a rhythm to be detected. Nevertheless, our analyses have demonstrated that significant changes in the statistical reliability of the rhythms across diagnoses are not dependent on the amount of movement produced, suggesting that actigraphy can be used to assess the statistical reliability of circadian sleep-wake cycles, regardless of the degree of activity exhibited by the patients.

A combination of the visual and motor subscales significantly predicted the goodness-of-fit of the circadian rhythms. The visual subscale score describes behaviors from absent visual startle at its lowest, through fixation and pursuit, to object recognition at its highest. This relationship is of particular interest since the master clock for circadian rhythms, the SCN, is itself timed by light inputs from the retina during the day, as well as melatonin from the pineal gland at night [29]. The more purposeful eye-movements of those scoring high on the visual subscale may allow for differing levels of light to reach the retina - perhaps through a greater ability to orient toward light or to maintain eye-opening for longer periods - and, consequently, result in a strengthening of the rhythm via the SCN. The predictive value of the visual subscale could, therefore, be considered to be consistent with our understanding of the biological generators of sleep-wake rhythmicity. This conclusion is necessarily speculative, however, since it is unclear whether high visual functioning is associated with a greater degree of orientation toward light or longer periods of eye-opening. Further investigation of this relationship will contribute to our understanding of the exogenous cues that drive circadian rhythms in VS/MCS patients.

A significant relationship was also found between the visual subscale of the CRS-R and the acrophase (time of maximal activity) of the rhythm, over and above the contribution of the other CRS-R subscales, or of the diagnosis of the patient. The relationship with acrophase reflects the tendency for patients with higher visual functioning to be most active later in the afternoon than patients with lower visual functioning (Visual Score >= 1, Mean acrophase (SD) 18:20 (three hours); Visual Score = 0, Mean acrophase (SD) 17:20 (two hours)). Consistent with this observation, exposure to higher levels of light has been associated with later peaks of activity in institutionalized individuals [41,42]. However, the activity peaks of healthy individuals occur earlier in the day than those observed in the patients here, typically between approximately 13:30p.m. and approximately 16:00p.m. [24]. It has been observed that the levels of light experienced by institutionalized patients are considerably lower than those of non-institutionalized individuals [42,43], and since the patients in the current study were residing on a hospital ward during the recording period, it is likely they were exposed to abnormally fluctuating levels of light compared with healthy individuals. Unfortunately, we were unable to record light levels alongside actigraphy; however, future studies investigating their contribution to the timing of activity of VS and MCS patients will be invaluable.

Since we inferred the circadian rhythms of patients from wrist actigraphy, it is likely that the recordings contain some levels of exogenous activity, perhaps from nurses moving the patient from bed to chair. Since these patients were all admitted to the same ward of the University Hospital of Liège as part of the same research protocol, they all received equivalent levels of care and were involved in the same assessments throughout the day - for example, behavioral tests, PET and MRI. As a result, the potential exogenous noise in the data would then be equally distributed across all patients. Our conclusions regarding the effects of diagnosis, etiology and behavioral profile on sleep-wake cycles, therefore, would remain valid despite this potential confound. The use of simultaneous video-recordings would allow for the exclusion of activity that is generated exogenously and would further validate our findings.

Some prescribed medications may also have an effect on actigraphy-detected circadian rhythms. For example, treatment for spasticity (for example, with baclofen) is common in VS/MCS patients and may increase the amount of movement that will be detected with actigraphy, while psychoactive medications (for example, amantadine) may also serve to exogenously modulate a patient's level of arousal. Caution in this regard is not limited to actigraphy, however, since psychoactive medications will also alter the resting EEG of a patient, thereby modulating the level of wakefulness that will be inferred from polysomnography. Due to differences in the wishes of families and physicians, a wide variety of medications are prescribed to VS and MCS patients (see Table 2 for details). As a result, it is not possible to statistically control for each of these drugs individually, nor for their many interactions. Nevertheless, there is no reason to believe that prescribed medications would systematically differ between VS and MCS groups due to the paucity of treatment recommendations for all patients with disorders of consciousness ([44]). Future controlled clinical trials are needed in order to provide insights into the effects of specific medications not only on circadian rhythmicity, but also on VS/MCS patient outcome in general.

**Table 2:** Patient etiology and prescribed daily medications.

Patient ID	Specific etiology	Daily medication dosage
1	Anoxia	1 × Clonazepam 2 mg 1 × Phenytoin 100 mg 1 × Clopidogrel 75 mg 1 × Acetylcysteine 600 mg 3 × Baclofen 25 mg 3 × Levetiracetam 500 mg

2	Anoxia	1 × Amantadine 100 mg 2 × Clonidine 150 mg 1 × Bisopropol 2.5 mg 1 × Paroxetine 20 mg 1 × Tetrazepam 50 mg 1 × Acetylcysteine 600 mg

3	Anoxia	2 × Lamotrigine 50 mg 1 × Levetiracetam 1,000 mg 3 × Diazepam 5 mg 3 × Baclofen 25 mg 1 × Esomeprazole 20 mg 1 × Domperidome 10 mg

4	Anoxia	1 × Vancomycin 2,000 mg 4 × Piperacillin 4,000 mg 4 × Amikacin 1,000 mg 1 × Enoxaparin Sodium 50 mg 3 × Paracetamol 1,000 mg 1 × Diazepam 10 mg 3 × Baclofen 15 mg 1 × Acetylcysteine 600 mg

5	Anoxia	3 × Valproic Acid 40 ml 3 × Diazepam 10 mg 2 × Terbutaline 5 mg 3 × Baclofen 10 mg 3 × Dantrolene 25 mg 1 × Enoxaparin Sodium 40 mg 2 × Fluconazole 50 mg 1 × Aspirin 160 mg

6	Anoxia	1 × Amantadine 100 mg 1 × Zolpidem 10 mg 1 × Esomeprazole 20 mg 1 × Trihexyphenidyl 4 mg

7	Tumor/Hemorrhage	2 × Lamotrigine 100 mg 1 × Aspirin 80 mg 1 × Omeprazole 20 mg 1 × Escitalopram 10 mg

8	Anoxia	1 × Aspirin 100 mg 3 × Tizanidine 4 mg 1 × Enoxaparin Sodium 40 mg 1 × Omeprazole 20 mg

9	Anoxia	1 × Esomeprazole 20 mg 2 × Valproic Acid 500 mg

10	Anoxia	1 × Esomeprazole 20 mg 3 × Valproic Acid 2 ml 2 × Levetiracetam 7.5 ml 1 × Enoxaparin Sodium 40 mg

11	Anoxia	3 × Baclofen 10 mg 1 × Omeprazole 10 mg 4 × Domperidone 1 mg 2 × Clonazepam 1 mg

12	Aneurysm	1 × Enoxaparin Sodium 40 mg

13	Anoxia	2 × Carbamazepine 200 mg

14	Trauma	1 × Valproic Acid 500 mg 1 × Lansoprazole 20 mg

15	Trauma	1 × Phenobarbital 100 mg 3 × Baclofen 10 mg

16	Trauma	1 × Valproic Acid 500 mg 1 × Bisoprolol 5 mg 3 × Piracetam 1,200 mg 3 × Baclofen 25 mg

17	Trauma	1 × Omeprazole 20 mg 2 × Flecainide 100 mg 2 × Levetiracetam 10 ml 3 × Baclofen 10 mg 2 × Sodium Valproate 600 ml 1 × Clonazepam 2 mg

18	Trauma	1 × Escitalopram 10 mg 4 × Alprazolam 250 mg 1 × Trazodone 100 mg 1 × Prothipendyl 80 mg

19	Trauma	2 × Baclofen 10 mg 1 × Paracetamol 500 mg 1 × Esomeprazole 40 mg 2 × Levetiracetam 7.5 ml

20	Trauma	1 × Bisopropol 2.5 mg 1 × Pantoprazole 20 mg 2 × Tizanidine 2 mg 1 × Enoxaparin Sodium 40 mg

21	Trauma	2 × Ranitidine 150 mg 3 × Baclofen 25 mg 1 × Enoxaparin Sodium 40 mg

22	Trauma	3 × Baclofen 25 mg 2 × Tizanidine 4 mg 1 × Enoxaparin Sodium 20 mg 1 × Amantadine 50 mg

23	Trauma	3 × Baclofen 25 mg 3 × Domperidome 10 mg 2 × Clonazepam 2.5 mg 1 × Promethazine 16 mg

24	Trauma	1 × Amantadine 100 mg 2 × Baclofen 10 mg 1 × Esomeprazole 20 mg 1 × Tizanidine 4 mg

25	Trauma	2 × Valproic Acid 7.5 ml 1 × Lamotrigine 25 mg 3 × Baclofen 10 mg 2 × Esomeprazole 20 mg 1 × Enoxaparin Sodium 20 mg

26	Trauma	1 × Esomeprazole 40 mg 3 × Clonazepam 2 mg 3 × Paracetamol 1,000 mg 2 × Levetiracetam 500 mg 3 × Benserazide 250 mg

27	Trauma	1 × Acetylcysteine 600 mg 1 × Esomeprazole 20 mg 1 × Baclofen 25 mg 1 × Atenolol 50 mg 1 × Enoxaparin Sodium 40 mg 1 × Glycopyrrolate 10 mg

28	Trauma	1 × Amantadine 100 mg 1 × Paroxetine 20 mg 3 × Domperidone 10 mg 1 × Esomeprazole 20 mg

29	Trauma	2 × Bisopropol 2.5 mg 1 × Esomeprazole 20 mg 1 × Amantadine 100 mg 3 × Metamizole 500 mg 3 × Meropenem 1,000 mg 3 × Ciprofloxacin 400 mg

30	Trauma	3 × Baclofen 25 mg 1 × Amantadine 100 mg 1 × Escitalopram 10 mg 1 × Enoxaparin Sodium 40 mg

31	Trauma	1 × Omeprazole 20 mg 1 × Ranitidine 300 mg 3 × Valproic Acid 2 ml 1 × Sertraline 2.5 mg 2 × Baclofen 5 ml

32	Trauma	2 × Carbamazepine 400 mg

33	Trauma	1 × Lansoprazole 30 mg 1 × Topiramate 150 mg 2 × Modafinil 100 mg 1 × Aniracetam 1,500 mg

34	Trauma	3 × Levetiracetam 500 mg 3 × Baclofen 10 mg 1 × Atenolol 50 mg 1 × Esomeprazole 20 mg 3 × Indometacin 50 mg

35	Trauma	2 × Ranitidine 150 mg 1 × Acetylcysteine 200 mg 3 × Baclofen 10 mg 1 × Escitalopram 10 mg 1 × Amantadine 100 mg 2 × Diclofenac 50 mg 1 × Enoxaparin Sodium 40 mg

36	Trauma	1 × Acetylcysteine 600 mg 1 × Esomeprazole 20 mg 1 × Baclofen 10 mg

37	Trauma	1 × Baclofen 10 mg 1 × Trazodone 25 mg 1 × Enoxaparin Sodium 40 mg

38	Anoxia	1 × Levetiracetam 3,000 mg 1 × Phenobarbital 100 mg 1 × Esomeprazole 40 mg 1 × Simvastatin 40 mg 1 × Aspirin 100 mg 1 × Escitalopram 10 mg

39	Anoxia	1 × Levothyroxine 25 mg 1 × Carbamazepine 200 mg

40	Anoxia	1 × Ranitidine 300 mg 3 × Phenobarbital 100 mg 3 × Baclofen 25 mg 5 × Lorazepam 2.5 mg 1 × Phenytoin 100 mg

41	Anoxia	1 × Amiodarone 200 mg 1 × Clopidogrel 75 mg 3 × Baclofen 25 mg 2 × Diltiazem 60 mg 1 × Trazodone 100 mg 1 × Lormetazepam 2 mg 2 × Prazepam 10 mg 1 × Enoxaparin Sodium 40 mg 2 × Acetylcysteine 400 mg 3 × Dantrolene 25 mg

42	Aneurysm	1 × Bisopropol 5 mg 3 × Baclofen 10 mg 1 × Levothyroxine 50 mg 1 × Prednisolone 5 mg

43	Meningitis	1 × Moxifloxacin 500 mg 1 × Levetiracetam 500 mg 2 × Ranitidine 150 mg 1 × Enoxaparin Sodium 40 mg

44	Anoxia	2 × Levetiracetam 1,000 mg 1 × Phenytoin 500 mg 6 × Valproic Acid 6.5 ml 1 × Lorazepam 1 mg 1 × Ranitidine 300 mg 2 × Enoxaparin Sodium 60 mg

45	Anoxia	3 × Valproic Acid 600 mg 1 × Ranitidine 150 mg

46	Cardio-respiratory Arrest	2 × Acetylcysteine 200 mg 1 × Enoxaparin Sodium 60 mg 1 × Ranitidine 10 ml

47	Anoxia	1 × Atenolol 25 mg 2 × Modafinil 100 mg

48	Anoxia	3 × Baclofen 10 mg 1 × Diazepam 5 mg 1 × Prazepam 5 mg 2 × Omeprazole 20 mg 1 × Levocetirizine 10 mg

49	Trauma	3 × Dantrolene 100 mg 3 × Carbamazepine 200 mg 3 × Baclofen 25 mg 1 × Omeprazole 20 mg 1 × Enoxaparin Sodium 40 mg

50	Trauma	3 × Baclofen 25 mg 1 × Pantoprazole 20 mg 3 × Dantrolene 100 mg 1 × Enoxaparin Sodium 40 mg

51	Trauma	3 × Baclofen 25 mg 1 × Trazodone 100 mg 1 × Enoxaparin Sodium 40 mg 2 × Levetiracetam 5 ml

52	Trauma	None

53	Trauma	4 × Paracetamol 500 mg 3 × Baclofen 10 mg 2 × Esomeprazole 20 mg 4 × Acetylcysteine 300 mg

54	Trauma	2 × Oxcarbazepine 450 mg 1 × Levetiracetam 5 ml 1 × Baclofen 25 mg 1 × Tizanidine 4 mg

55	Trauma	2 × Levetiracetam 100 mg 2 × Ranitidine 150 mg 2 × Lamotrigine 25 mg 3 × Baclofen 25 mg 1 × Enoxaparin Sodium 40 mg

A final caveat is that the apparent absence of reliable circadian rhythms in some of our patients may be a result of a lack of sensitivity of the actigraphy method, rather than the true absence of those rhythms. While wrist actigraphy has been validated for sleep assessment in patients with C5 to C7 tetraplegia [23], these patients are nevertheless capable of small but purposeful wrist movements. Patients in the VS, however, are by definition unable to produce purposeful movements, although spontaneous movements are common. Similarly, due to the heterogeneity of brain injuries of these patients, it is not clear whether the presence of actigraphy-detected waking is necessarily indicative of concurrent cerebral waking. Future validation of the relationship between polysomnography and actigraphy measures of sleeping and waking in VS and MCS patients is needed in order to fully characterize the nature of their circadian rhythms.

## Conclusions

Our analyses indicate a greater impairment of circadian sleep-wake cycling in patients in the VS compared with those in the MCS, and in those with non-TBI compared with TBI. The significant differences observed between VS and MCS patients support the conclusion that these are diagnostically distinct entities. However, they also suggest that despite periods of eye-closure and eye-opening, sleep-wake cycles are not necessarily present despite the clinical criteria for these conditions [5-7]. Wrist actigraphy is considerably less expensive and less invasive than other forms of sleep-wake monitoring and may, therefore, provide a reliable means of determining the extent to which these cycles are preserved in individual patients. These recordings could also allow clinicians and researchers to identify the time of day in which a patient is most active, in order to schedule behavioral and/or neuroimaging assessments for a time that maximizes the likelihood of detecting an appropriate response (see [26]). Future validation of the relationship between actigraphy and polysomnography measures of sleeping/waking in VS and MCS patients will allow for a more complete understanding of the physiological nature of these circadian rhythms. Follow-up studies will also determine the prognostic utility of wrist actigraphy for VS and MCS patients.

## Abbreviations

ARAS: ascending reticular activating system; CRS-R: Coma Recovery Scale Revised; DTI: diffusion tensor imaging; EEG: electroencephalography; MCS: Minimally Conscious State; MRI: magnetic resonance imaging; non-TBI: non-traumatic brain injuries; PET: positron emission tomography; SCN: suprachiasmatic nuclei; TBI: traumatic brain injuries; UWS: Unresponsive Wakefulness Syndrome; VS: Vegetative State.

## Competing interests

The authors declare that they have no competing interests.

## Authors' contributions

DC designed and conducted the analyses and wrote the manuscript. AT, AD, MAB, OG and AV collected all data and contributed to the final manuscript. JCN contributed to the analyses and the final manuscript. TAB, AMO and SL provided conceptual input and contributed to the final manuscript. All authors approved the final version of the manuscript.

## Pre-publication history

The pre-publication history for this paper can be accessed here:

http://www.biomedcentral.com/1741-7015/11/18/prepub

## References

[B1] Laureys S Celesia GG Cohadon F Lavrijsen J Leon-Carrion J Sannita WG Sazbon L Schmutzhard E von Wild KR Zeman A Dolce G Unresponsive wakefulness syndrome: a new name for the vegetative state or apallic syndrome BMC Med 2010 8 68 10.1186/1741-7015-8-68 21040571 PMC2987895

[B2] Laureys S The neural correlate of (un)awareness: lessons from the vegetative state Trends Cogn Sci 2005 9 556 559 10.1016/j.tics.2005.10.010 16271507

[B3] Jennett B Plum F Persistent vegetative state after brain damage. A syndrome in search of a name Lancet 1972 1 734 737 4111204 10.1016/s0140-6736(72)90242-5

[B4] Giacino JT Kalmar K Whyte J The JFK Coma Recovery Scale-Revised: measurement characteristics and diagnostic utility Arch Phys Med Rehabil 2004 85 2020 2029 10.1016/j.apmr.2004.02.033 15605342

[B5] The Multi-Society Task Force on PVS Medical aspects of the persistent vegetative state (1) N Engl J Med 1994 330 1499 1508 7818633 10.1056/NEJM199405263302107

[B6] Royal College of Physicians The permanent vegetative state. Review by a working group convened by the Royal College of Physicians and endorsed by the Conference of Medical Royal Colleges and their faculties of the United Kingdom J R Coll Physicians Lond 1996 30 119 121 Updated 2003 8709056 PMC5401536

[B7] Giacino JT Ashwal S Childs N Cranford R Jennett B Katz DI Kelly JP Rosenberg JH Whyte J Zafonte RD Zasler ND The minimally conscious state: definition and diagnostic criteria Neurology 2002 58 349 353 10.1212/WNL.58.3.349 11839831

[B8] Owen AM Coleman MR Boly M Davis MH Laureys S Pickard JD Detecting awareness in the vegetative state Science 2006 313 1402 10.1126/science.1130197 16959998

[B9] Monti MM Vanhaudenhuyse A Coleman MR Boly M Pickard JD Tshibanda L Owen AM Laureys S Willful modulation of brain activity in disorders of consciousness N Engl J Med 2010 362 579 589 10.1056/NEJMoa0905370 20130250

[B10] Cruse D Chennu S Chatelle C Bekinschtein TA Fernández-Espejo D Pickard JD Laureys S Owen AM Bedside detection of awareness in the vegetative state - a cohort study Lancet 2011 378 2088 2094 10.1016/S0140-6736(11)61224-5 22078855

[B11] Refinetti R Circadian Physiology 2006 Boca Raton, FL: Taylor & Francis Group

[B12] Ancoli-Israel S Cole R Alessi C Chambers M Moorcroft W Pollak CP The role of actigraphy in the study of sleep and circadian rhythms Sleep 2003 26 342 392 12749557 10.1093/sleep/26.3.342

[B13] Landsness E Bruno MA Noirhomme Q Riedner B Gosseries O Schnakers C Massimini M Laureys S Tononi G Boly M Electrophysiological correlates of behavioural changes in vigilance in vegetative state and minimally conscious state Brain 2011 134 2222 2232 10.1093/brain/awr152 21841201 PMC3155704

[B14] Isono M Wakabayashi Y Fujiki MM Kamida T Kobayashi H Sleep cycle in patients in a state of permanent unconsciousness Brain Inj 2002 16 705 712 10.1080/02699050210127303 12167195

[B15] Fukudome Y Abe I Saku Y Matsumura K Sadoshima S Utunomiya H Fujishima M Circadian blood pressure in patients in a persistent vegetative state Am J Physiol 1996 270 R1109 1114 8928913 10.1152/ajpregu.1996.270.5.R1109

[B16] Pattoneri P Tirabassi G Pela G Astorri E Mazzucchi A Borghetti A Circadian blood pressure and heart rate changes in patients in a persistent vegetative state after traumatic brain injury J Clin Hypertens (Greenwich) 2005 7 734 739 10.1111/j.1524-6175.2005.04780.x 16330896 PMC8109359

[B17] Guan J You C Liu Y Zhang R Wang Z Characteristics of infradian and circadian rhythms in the persistent vegetative state J Int Med Res 2011 39 2281 2287 22289544 10.1177/147323001103900625

[B18] Oksenberg A Arons E Sazbon L Mizrahi A Radwan H Sleep-related erections in vegetative state patients Sleep 2000 23 953 957 11083604

[B19] Candelieri A Cortese MD Dolce G Riganello F Sannita WG Visual pursuit: within-day variability in the severe disorder of consciousness J Neurotrauma 2011 28 2013 2017 10.1089/neu.2011.1885 21770758

[B20] Bekinschtein TA Golombek DA Simonetta SH Coleman MR Manes FF Circadian rhythms in the vegetative state Brain Inj 2009 23 915 919 10.1080/02699050903283197 20100128

[B21] de Souza L Benedito-Silva AA Pires ML Poyares D Tufik S Calil HM Further validation of actigraphy for sleep studies Sleep 2003 26 81 85 12627737 10.1093/sleep/26.1.81

[B22] Berger AM Wielgus KK Young-McCaughan S Fischer P Farr L Lee KA Methodological challenges when using actigraphy in research J Pain Symptom Manage 2008 36 191 199 10.1016/j.jpainsymman.2007.10.008 18400460 PMC2542506

[B23] Spivak E Oksenberg A Catz A The feasibility of sleep assessment by actigraph in patients with tetraplegia Spinal Cord 2007 45 765 770 10.1038/sj.sc.3102040 17339889

[B24] Brown A Smolensky M D'Alonzo G Redmond D Conrad E Hsi B Circadian rhythm in human activity objectively quantified by actigraphy Prog Clin Biol Res 1990 341A 77 83 2217296

[B25] Girardin J-L Kripke DF Ancoli-Israel S Klauber MR Sepulveda RS Mowen M-A Assmus JD Langer RD Circadian sleep, illumination, and activity patterns in women: influences of aging and time reference Physiol Behav 2000 68 347 352 10.1016/S0031-9384(99)00186-9 10716544

[B26] Bekinschtein T Cologan V Dahmen B Golombek D You are only coming through in waves: wakefulness variability and assessment in patients with impaired consciousness Prog Brain Res 2009 177 171 189 19818901 10.1016/S0079-6123(09)17712-9

[B27] De Weer AS Da Ros M Berré J Mélot C Goldman S Peigneux P Environmental influences on activity patterns in altered states of consciousness Eur J Neurol 2011 18 1432 1434 10.1111/j.1468-1331.2011.03477.x 21771202

[B28] Nelson W Tong YL Lee JK Halberg F Methods for cosinor-rhythmometry Chronobiologia 1979 6 305 323 548245

[B29] Saper CB Scammell TE Lu J Hypothalamic regulation of sleep and circadian rhythms Nature 2005 437 1257 1263 10.1038/nature04284 16251950

[B30] Schiff ND Central thalamic contributions to arousal regulation and neurological disorders of consciousness Ann N Y Acad Sci 2008 1129 105 118 10.1196/annals.1417.029 18591473

[B31] Fernandez-Espejo D Junque C Bernabeu M Roig-Rovira T Vendrell P Mercader JM Reductions of thalamic volume and regional shape changes in the vegetative and the minimally conscious states J Neurotrauma 2010 27 1187 1193 10.1089/neu.2010.1297 20392136

[B32] Fernández-Espejo D Bekinschtein T Monti MM Pickard JD Junque C Coleman MR Owen AM Diffusion weighted imaging distinguishes the vegetative state from the minimally conscious state NeuroImage 2011 54 103 112 10.1016/j.neuroimage.2010.08.035 20728553

[B33] Adams JH Graham DI Murray LS Scott G Diffuse axonal injury due to nonmissile head-injury in humans - an analysis of 45 cases Ann Neurol 1982 12 557 563 10.1002/ana.410120610 7159059

[B34] Kinney HC Samuels MA Neuropathology of the persistent vegetative state. A review J Neuropathol Exp Neurol 1994 53 548 558 10.1097/00005072-199411000-00002 7964896

[B35] Adams JH Jennett B McLellan DR Murray LS Graham DI The neuropathology of the vegetative state after head injury J Clin Pathol 1999 52 804 806 10.1136/jcp.52.11.804 10690167 PMC501589

[B36] Jennett B Adams JH Murray LS Graham DI Neuropathology in vegetative and severely disabled patients after head injury Neurology 2001 56 486 490 10.1212/WNL.56.4.486 11222792

[B37] Adams JH Duchen LW Greenfield's Neuropathology 1992 5 New York, New York: Oxford University Press

[B38] Adams JH Graham DI Jennett B The neuropathology of the vegetative state after an acute brain insult Brain 2000 123 1327 1338 10.1093/brain/123.7.1327 10869046

[B39] Kinney HC Korein J Panigrahy A Dikkes P Goode R Neuropathological findings in the brain of Karen Ann Quinlan. The role of the thalamus in the persistent vegetative state N Engl J Med 1994 330 1469 1475 10.1056/NEJM199405263302101 8164698

[B40] Veasey SC Davis CW Fenik P Zhan G Hsu YJ Pratico D Gow A Long-term intermittent hypoxia in mice: protracted hypersomnolence with oxidative injury to sleep-wake brain regions Sleep 2004 27 194 201 15124711 10.1093/sleep/27.2.194

[B41] Shochat T Martin J Marler M Ancoli-Israel S Illumination levels in nursing home patients: effects on sleep and activity rhythms J Sleep Res 2000 9 373 379 10.1046/j.1365-2869.2000.00221.x 11386204

[B42] Ancoli-Israel S Martin JL Kripke DF Marler M Klauber MR Effect of light treatment on sleep and circadian rhythms in demented nursing home patients J Am Geriatr Soc 2002 50 282 289 10.1046/j.1532-5415.2002.50060.x 12028210 PMC2764401

[B43] Campbell SS Kripke DF Gillin JC Hrubovcak JC Exposure to light in healthy elderly subjects and Alzheimer's patients Physiol Behav 1988 42 141 144 10.1016/0031-9384(88)90289-2 3368532

[B44] Giacino J Whyte J The vegetative and minimally conscious states: current knowledge and remaining questions J Head Trauma Rehabil 2005 20 30 50 10.1097/00001199-200501000-00005 15668569

