# Correction to: Actigraphy assessments of circadian sleep-wake cycles in the Vegetative and Minimally Conscious States

**DOI:** 10.1186/s12916-018-1139-y

**Published:** 2018-08-11

**Authors:** D. Cruse, A. Thibaut, A. Demertzi, J. C. Nantes, M. A. Bruno, O. Gosseries, A. Vanhaudenhuyse, T. A. Bekinschtein, A. M. Owen, S. Laureys

**Affiliations:** Brain and Mind Institute, University of Western Ontario, 1151 Richmond Street, London, ON N6A 3K7 Canada

## Correction

The original article [1] contains an error affecting the actigraphy time-stamps throughout the article, particularly in Table 1.

**Table 1:** Demographics and circadian rhythm fits for all patients

Patient ID	Gender	Age (Years)	Post-Ictus (Months)	Diagnosis	Etiology	CRS-R	Mesor	Acrophase	Amplitude	Sig. Fit?
1	M	53	40	MCS	Non-TBI	11	9.56	10:57	6.7	Yes
2	M	31	22	MCS	Non-TBI	13	8.67	15:44	10.46	Yes
3	W	30	78	MCS	Non-TBI	9	15.84	9:45	13.58	Yes
4	M	31	16	MCS	Non-TBI	7	5.51	13:27	4.63	Yes
5	M	27	50	MCS	Non-TBI	9	31.01	9:43	9.94	No
6	W	36	17	MCS	Non-TBI	13	21.25	13:10	15.87	Yes
7	M	34	35	MCS	Non-TBI	12	26.4	11:35	28.34	Yes
8	W	63	3	MCS	Non-TBI	13	6.32	10:36	6.77	Yes
9	M	57	12	MCS	Non-TBI	7	12.89	11:26	9.4	Yes
10	M	66	2	MCS	Non-TBI	10	34.18	16:25	26	Yes
11	M	11	48	MCS	Non-TBI	13	32.4	13:05	35.79	Yes
12	W	43	3	MCS	Non-TBI	6	9.12	11:49	8.76	Yes
13	W	34	256	MCS	Non-TBI	12	20.63	12:40	15.36	Yes
14	M	30	106	MCS	TBI	14	49.2	14:18	41.91	Yes
15	W	21	1	MCS	TBI	10	17.23	11:57	12.52	Yes
16	M	46	17	MCS	TBI	11	42.8	16:46	48.47	Yes
17	M	30	27	MCS	TBI	10	20.71	14:12	17.98	Yes
18	M	30	13	MCS	TBI	9	54.24	11:16	22.6	Yes
19	M	24	10	MCS	TBI	10	114.11	12:50	65.35	Yes
20	W	75	9	MCS	TBI	9	8.57	11:06	9.55	Yes
21	W	34	99	MCS	TBI	12	18.22	9:46	6.64	No
22	W	27	41	MCS	TBI	11	15.02	17:05	16.01	Yes
23	M	24	88	MCS	TBI	11	18.54	13:49	14.62	No
24	M	44	287	MCS	TBI	9	5.25	13:37	4.83	Yes
25	W	30	4	MCS	TBI	9	10.41	11:29	13.41	Yes
26	M	34	33	MCS	TBI	8	27.22	12:30	27.89	No
27	M	23	10	MCS	TBI	10	8.47	10:38	7.74	Yes
28	M	27	37	MCS	TBI	13	40.74	14:53	34.18	Yes
29	M	61	4	MCS	TBI	10	21.8	15:41	19.72	Yes
30	M	24	24	MCS	TBI	11	88.98	16:18	56.45	No
31	M	23	66	MCS	TBI	16	14.09	11:24	18.96	No
32	M	21	38	MCS	TBI	8	10.09	9:55	7.06	Yes
33	M	30	109	MCS	TBI	10	35.15	14:26	21.87	Yes
34	W	24	21	MCS	TBI	10	15.45	12:58	18.08	Yes
35	M	36	4	MCS	TBI	11	6.49	10:10	5.53	Yes
36	M	65	22	MCS	TBI	7	11.3	10:13	15.14	Yes
37	M	21	5	MCS	TBI	7	9.69	7:41	7.9	Yes
38	W	66	0	VS	Non-TBI	3	4.56	9:43	5.24	Yes
39	M	35	220	VS	Non-TBI	7	11.01	13:20	10.24	Yes
40	M	30	24	VS	Non-TBI	6	25.15	9:28	14.67	Yes
41	W	48	15	VS	Non-TBI	5	8.5	11:14	10.53	Yes
42	W	67	45	VS	Non-TBI	5	15.46	12:29	12.23	Yes
43	M	53	1	VS	Non-TBI	5	9.53	10:53	7.92	Yes
44	M	34	17	VS	Non-TBI	7	5.68	11:43	2.79	Yes
45	W	41	56	VS	Non-TBI	5	14.39	11:35	14.49	Yes
46	W	48	4	VS	Non-TBI	4	4.78	14:02	3.44	No
47	M	48	30	VS	Non-TBI	6	2.31	11:19	2.03	Yes
48	M	36	66	VS	Non-TBI	5	7.34	11:40	6.88	Yes
49	M	34	43	VS	TBI	6	10.84	7:47	7.87	Yes
50	W	30	18	VS	TBI	4	6.49	10:56	8.07	Yes
51	M	21	7	VS	TBI	7	8.58	12:15	8.29	Yes
52	M	35	290	VS	TBI	8	57.65	17:10	36.56	No
53	M	21	8	VS	TBI	6	9.95	13:22	6.45	Yes
54	M	13	1	VS	TBI	6	5.78	15:24	3.12	No
55	M	25	15	VS	TBI	5	10.02	10:19	9.05	Yes

The final column indicates whether the circadian rhythm fit was significant or not. *MCS* Minimally Conscious State, *TBI* Traumatic Brain Injury, *VS* Vegetative State

All data for this manuscript were collected in Liège, Belgium, and exported and analysed in London, Ontario. The authors have recently discovered that a software error occurred during data export that caused all actigraphy time-stamps to be shifted by exactly 6-hours due to the time-zone difference between Belgium and Ontario. As a result, the acrophase values reported in the original Table 1 should all be read as occurring 6-hours earlier in the day as displayed in the corrected Table 1 ahead (for example, if an acrophase is reported as 16:57 in Table 1, it should be read as 10:57). The corrected Table 1 appears as within this Erratum.

Importantly, as this software error added only a constant value to each time-stamp of each patient data set, no analyses or results are affected.
